# CONSTANS-Like 9 (OsCOL9) Interacts with Receptor for Activated C-Kinase 1(OsRACK1) to Regulate Blast Resistance through Salicylic Acid and Ethylene Signaling Pathways

**DOI:** 10.1371/journal.pone.0166249

**Published:** 2016-11-09

**Authors:** Hao Liu, Shuangyu Dong, Dayuan Sun, Wei Liu, Fengwei Gu, Yongzhu Liu, Tao Guo, Hui Wang, Jiafeng Wang, Zhiqiang Chen

**Affiliations:** 1 National Engineering Research Center of Plant Space Breeding, South China Agricultural University, Guangzhou, 510642, China; 2 Plant Protection Research Institute Guangdong Academy of Agricultural Sciences/Guangdong Provincial Key Laboratory of High Technology for Plant Protection, Guangzhou, 510640, China; Texas A&M University College Station, UNITED STATES

## Abstract

In a previous transcriptome analysis of early response genes in rice during *Magnaporthe oryzae* infection, we identified a CONSTANS-like (*COL*) gene *OsCOL9*. In the present study, we investigated the functional roles of *OsCOL9* in blast resistance. *OsCOL9* belonged to group II of the COL protein family, and it contained a BB-box and a C-terminal CCT (CONSTANS, COL and TOC1) domain. *OsCOL9* was found in the nucleus of rice cells, and it exerted transcriptional activation activities through its middle region (MR). *Magnaporthe oryzae* infection induced *OsCOL9* expression, and transgenic *OsCOL9* knock-out rice plants showed increased pathogen susceptibility. *OsCOL9* was a critical regulator of pathogen-related genes, especially *PR1b*, which were also activated by exogenous salicylic acid (SA) and 1-aminocyclopropane-1-carboxylicacid (ACC), the precursor of ethylene (ET). Further analysis indicated that *OsCOL9* over-expression increased the expressions of phytohormone biosynthetic genes, *NPR1*, *WRKY45*, *OsACO1* and *OsACS1*, which were related to SA and ET biosynthesis. Interestingly, we found that OsCOL9 physically interacted with the scaffold protein OsRACK1 through its CCT domain, and the *OsRACK1* expression was induced in response to exogenous SA and ACC as well as *M*. *oryzae* infection. Taken together, these results indicated that the COL protein OsCOL9 interacted with OsRACK1, and it enhanced the rice blast resistance through SA and ET signaling pathways.

## Introduction

During their coevolution with pathogens, plants have developed multiple layers of sophisticated mechanisms against invading pathogens. The first layer of defense is PAMP-triggered immunity (PTI), which relies on recognition of microbial or pathogen-associated molecular patterns (MAMPs or PAMPs) through interaction with their cognate pattern recognition receptors (PRRs) [1]. The second layer of defense functions *via* recognition of variable pathogen-derived effector molecules [avirulence (AVR) proteins] by effector-triggered immunity (ETI) [2]. ETI facilitates more rapid and stronger immune responses than PTI, efficiently inhibiting and limiting pathogen spreading.

PTI and ETI usually activate a similar set of defense responses, including ROS production, anion channel opening, MAPK activation, phytoalexin production and defense gene expression. Signal transduction and fine-tuning of gene expression are required to regulate these defense mechanisms [3]. A number of transcription factors, such as WRKY, NAC and bZIP, from different plant species play important roles in host-pathogen interactions [4–6]. These transcription factors function either as positive or negative regulators in defense responses. For example, transgenic plants over-expressing *OsWRKY13*, *OsWRKY31*, *OsWRKY45* or *OsWRKY53* show enhanced basal resistance to *M*. *oryzae*, indicating their important roles in blast resistance [7–10]. *OsWRKY45* regulates rice blast resistance of the CC-NB-LRR protein Pb1 through protein-protein interactions, indicating that *OsWRKY45* may function in both ETI and PTI [11]. Furthermore, *ANAC019* and *ANAC055* are crucial regulators of the phytohormone-induced expression of defense genes [12]. This evidence indicates that transcription factors regulate and fine-tune defense-related gene expression as well as metabolite synthesis during pathogen attack.

CONSTANS-like (*COL*) genes contain two conserved domains [BB-box and CCT (CONSTANS, COL and TOC1)] that play key roles in photoperiodic flowering, abiotic stress and biotic stress [13–15]. Global expression profiling analysis indicates that *COL9* is involved in fungal defense and *COL4* is involved in the jasmonic acid (JA) and abscisic acid (ABA) metabolic pathways [16,17]. For instance, *MaCOL1* expression in *Arabidopsis* is induced by pathogen infection stress [18]. This suggests a possible role of the *COL* genes in response to pathogen attack. However, the biological functions of these *COL* genes in disease resistance remain unknown.

In this study, we characterized the role of *OsCOL9* in rice blast resistance. We found that OsCOL9 was a nuclear protein and functioned as a transcriptional activator through its MR domain. *OsCOL9* expression was increased upon pathogen infection, and its over-expression enhanced the resistance against *M*. *oryzae*. Finally, OsCOL9 interacted with OsRACK1 and modulated phytohormone biosynthetic genes expression. These results provided evidence that *OsCOL9* was a transcriptional activator that regulated rice blast resistance by phytohormone biosynthesis. This study further extended our understanding of the role of *COL* genes in disease resistance.

## Materials and Methods

### Plant materials and treatments

The *Oryza sativa japonica Pik-H4* near isogenic lines (*Pik-H4 NILs*) were used as wild-type (WT) plants in this study. These plants contain the *Pik-H4* resistance gene allelic at the *Pik* locus [19]. In addition, a susceptible *O*. *sativa japonica* Lijiangxintuanheigu (LTH) strain was used as control. For fungal infections, we isolated the *M*. *oryzae* race GDR2 from diseased nurseries and fields at Yangjing city(111.95°E, 21.85°N) by Dayuan Sun, there is no specific permission was required and this work was supported by the Plant Protection Research Institute Guangdong Academy of Agricultural Sciences/Guangdong Provincial key Laboratory of High Technology for Plant Protection. This *M*. *oryzae* race is friendly to human and animals, which is just compatible with the rice strain that not contains the resistance gene *Pik-H4*.

For inoculation of rice blast fungus, we used fourth-leaf-stage rice seedlings grown under natural light in a greenhouse at 26°C. Freshly prepared *M*. *oryzae* conidia (1×10^5^ conidia/ml, containing 0.02% v/v gelatin) were sprayed onto the rice leaves using an air sprayer. Inoculated plants were kept in a humidity chamber at 28°C and rice leaves were harvested for extraction of RNA at 0, 12, 24, 36, 48, 60 and 72 h after inoculation. The concentrations of JA (100 μM), SA (100μM), ACC (1 mM) and ABA (100μM) were applied to whole rice plants at the fourth-leaf stage. The treated plants were immediately covered with a transparent lid and the leaves were collected after treatments [20].

### Subcellular localization analysis

The full-length *OsCOL9* cDNA insert lacking a stop codon was amplified by PCR using GFP-F/R primers (S1 Table). Amplified fragments were *Xba*I/*Bam*HI digested and cloned between the CaMV35S promoter and GFP sites of pUC-GFP, a pUC18 derivative [21]. The resulting GFP fusion constructs and the nuclear localization marker [22] were then transiently co-expressed in rice protoplasts [23] which were then examined by laser-scanning confocal microscopy (LSM 780, Carl Zeiss, Jena, Germany). Excitation/emission wavelengths were 514/535 590 nm for GFP and 543/565 615 nm for the mCherry construct.

### Determination of transcriptional activity and the activation domain of OsCOL9

The OsCOL9 protein has three conserved COL domains. To determine its transcriptional activity and map its transcriptional activation domain, various regions of the OsCOL9 protein coding sequence, including the BB-box domain (amino acids 1–150), the CCT domain (amino acids 301–422), the middle region (MR) (amino acids 151–300) and the full-length OsCOL9 were amplified by PCR with specific primers (S1 Table) and cloned into *Nde*I/*Bam*HI-digested pGBKT7 vector. The resulting vectors, the positive control OsBIHD1, and the negative control pGBKT7 (empty vector) were transformed into yeast strain Y2H Gold strain. Positive transformants were grown on SD/-Trp plates and SD/-Trp/-His/-Ade plates (with 100 mM 3-AT) for 3 days at 30°C. X-α-gal was used to identify the transcription activation activity of the OsCOL9 protein domains.

### Total RNA extraction, quantitative real-time PCR analysis of gene expression

Total RNA was extracted from 100 mg of fourth-leaf-stage rice seedlings with TRIZOL Reagent (Invitrogen, Beijing, China) and reversed-transcribed into cDNA using PrimeScript RT reagent Kit (Takara, Dalian, China) according to the manufacturer’s instructions. The reverse transcribed cDNA samples were used for real-time PCR on an ABI Step One plus real-time PCR detection system. The specific primers used for quantitative real-time PCR analysis are shown in S2 Table. Quantitative real-time PCR reactions were performed in a 25 μl volume containing 12.5 μl of SYBR Premix ExTaq (TaKaRa, Dalian, China). Differences in gene expression, expressed as fold change relative to control, were calculated using the 2^-ΔΔCT^ method. Each measurement was carried out in triplicate, and the error bars represent SE of the mean of fold changes for the three biological replicates.

### Generation of the *OsCOL9-OX* and *oscol9-ko* transgenic plants

The full-length OsCOL9 cDNA was isolated by RT-PCR from the leaves of fourth-leaf-stage rice plants using the cDNA primers encompassing translation start and stop codons (S1 Table). This cDNA insert digested with *Hind* I and *Bam*HI was then cloned between the maize ubiquitin promoter and the Nos terminator of the plant expression vector pOX [24] containing the hygromycin resistance gene as selection maker. CRISPR/Cas9 technology was used to generate *oscol9-ko* plants. As reported by Ma et al [25], a 20 bp DNA fragment (including PAM) of the first exon of the *OsCOL9* nucleotide sequence was fused with U6a-gRNA box. The resulting DNA insert was digested with *Bsa*I and inserted into the pYLCRISPR/Cas9PUbi-H vector. pOX-OsCOL9 and pYLCRISPR/Cas9-OsCOL9 were then introduced into agrobacterium strain EHA105 and transformed into wild-type (*Pik-H4 NILs*) calli as described previously by Hiei [26]. Transgenic rice plants were regenerated from the transformed calli in selection media containing 50 mg L^-1^ hygromycin and 250 mg L^-1^ cefotaxime. *OsCOL9* expression in the transgenic rice plants was confirmed by target site sequencing and qRT-PCR.

### Yeast-two hybrid assay

The OsCOL9 BBOX_1-150aa_ and CCT_301-422aa_ were cloned into the BD plasmid pGBKT7 by homologous recombination in yeast strain AH109. Yeast cells containing the resulting constructs were used as bait to screen the interacting-proteins from a rice yeast two-hybrid c-DNA library, according to the manufacturer’s instructions of Clontech yeast two-hybrid handbook. The transformed yeast cells were cultured on SD/-Trp/-Leu and SD/-Trp/-Leu/-His+3AT plates and results were scored after 3 days incubation at 30°C.

### Bimolecular fluorescence complementation (BiFC) assay

For BiFC assays, the coding regions of *OsCOL9* and *OsRACK1* were cloned into the NheI /AgeI sites of the BiFC vectors pUC-NE1L2L-nsI and pUC-CE1RL2R-nsI to generate OsRACK1-nYFP and OsCOL9-cYFP constructs, respectively. The recombinant constructs in pairs were co-transfected into rice protoplasts, and fluorescent signals were examined by confocal microscopy Carl Zeiss LSM780.

### GST pull down assay

Full-length domains of the OsCOL9 CCT cDNA sequence with stop codon and the full length *OsRACK1*cDNA were cloned into BamHI/*EcoR*I sites of pGEX6p-1 and pET28, respectively. OsCOL9 CCT-GST and OsRACK1-His fusion proteins expression were induced using 0.5 mM isopropyl β-D-thiogalactoside (IPTG) for 12 h at 37°C in *Escherichia coli* strain BL21. His-tagged proteins were incubated with GST-COL9 CCT or GST alone bound to glutathione beads. After 4 h of incubation at 4°C, the beads were washed four times with GST binding buffer (PBS, pH 7.2). Components bound to the beads were eluted by boiling in SDS sample buffer and were then separated on an SDS-PAGE gel and immunoblotted with anti-His and anti-GST antibodies.

## Results

### OsCOL9 is localized to the nucleus and activates transcription through its MR domain

**Fig 1:**
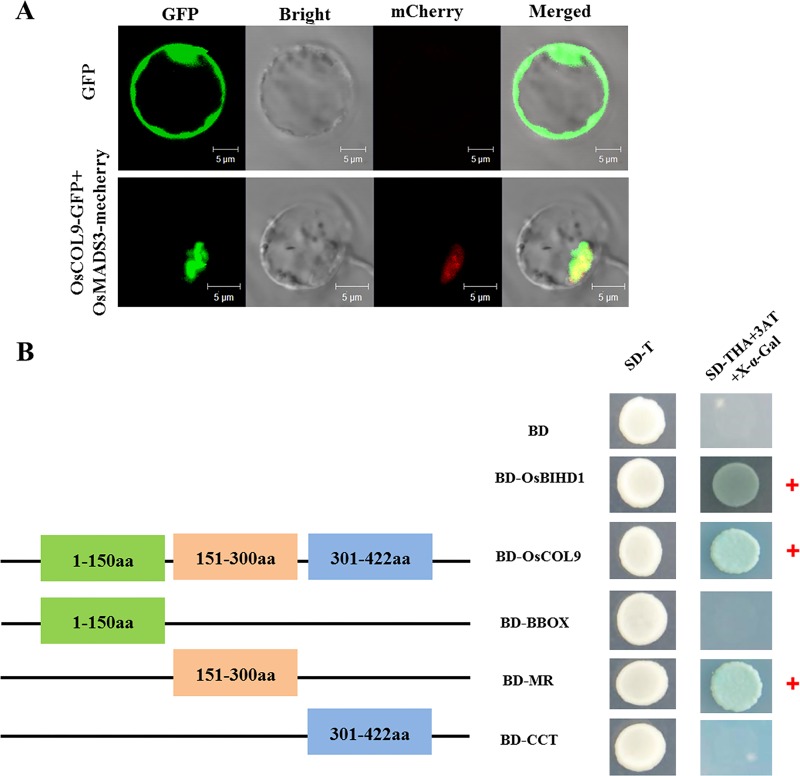
Nuclear localization and transcriptional activity of OsCOL9. (A) Subcellular localization of OsCOL9 in rice protoplast cells. 35S:GFP served as control and 35S:OsMADS3-mCherry served as a nuclear marker. Cell fluorescence was observed using confocal microscope at 12 h post-transformation. OsCOL9-GFP signals are green, and the nuclear signals are red. Merged images of GFP, mCherry and bright-fields are shown. Scale bar is 5 μm. (B) Transcriptional activation analysis of OsCOL9 and three truncated mutants (B-box, MR and CCT) fused with the yeast GAL4 DNA-binding domain. Full-length OsCOL9 and the truncated mutants were fused with pGBKT7, and the constructs were transformed into Y2H Gold yeast cells. Transcriptional activation activity was determined according to the ability to activate the expression of His, Ade and X-α-Gal reporter genes in yeast. BD-OsCOL9 and BD-MR transformants were selected on SD/-Trp/-His/-Ade plates containing X-α-Gal and 100 mM 3-AT. OsCOL9 and its MR domain contained the transcriptional activity by a positive (+) reaction in the X-α-Gal assay.

*OsCOL9* belonged to subgroup II of *COL* gene family in *O*. *sativa* and contained one BB-box and one CCT domain (S1 Fig). To determine the subcellular localization of OsCOL9, the protein was fused to green fluorescent protein (GFP) under the control of the constitutive CaMV35S promoter. The resulting vectors (35S:OsCOL9-GFP, 35S:GFP) and a nuclear localization marker OsMADS3-mCherry were transiently co-transfected into rice protoplast cells with the PEG-mediated procedure. The OsCOL9-GFP fusion protein and the nuclear localization marker signals merged in the nucleus of the rice protoplasts. The signals of control 35S-GFP were observed throughout the cells, including the cytoplasm and nucleus (Fig 1A). These results indicated that OsCOL9 was localized to the nucleus.

To further examine whether OsCOL9 had transcriptional activation activity and which part of OsCOL9 protein held the activity *in vivo*, we examined constructs containing various regions of the OsCOL9 protein using a yeast two-hybrid assay (Fig 1B). These regions included the BB-box domain (amino acids 1–150), the CCT domain (amino acids 301–422), the middle region (MR) (amino acids 151–300) and the full-length OsCOL9. These regions were respectively fused to the *GAL4* DNA-binding domain in the pGBKT7 vector, and then transformed into Y2H Gold yeast cells. All the transformants containing pGBKT7-OsCOL9 or pGBKT7-MR grew well on SD/-Trp and SD/-Trp/-Ade/-His +100 mM 3-AT +X-gal plates, and these yeast cells were stained with blue in X-gal solution. In contrast, the yeast cells containing pGBKT7-BB-box, pGBKT7-CCT or the negative control pGBKT7 grew only on SD/-Trp plates (Fig 1B). These results demonstrated that OsCOL9 held the transcriptional activation activity through its MR domain.

### *M*. *oryzae* up-regulates *OsCOL9* expression

**Fig 2:**
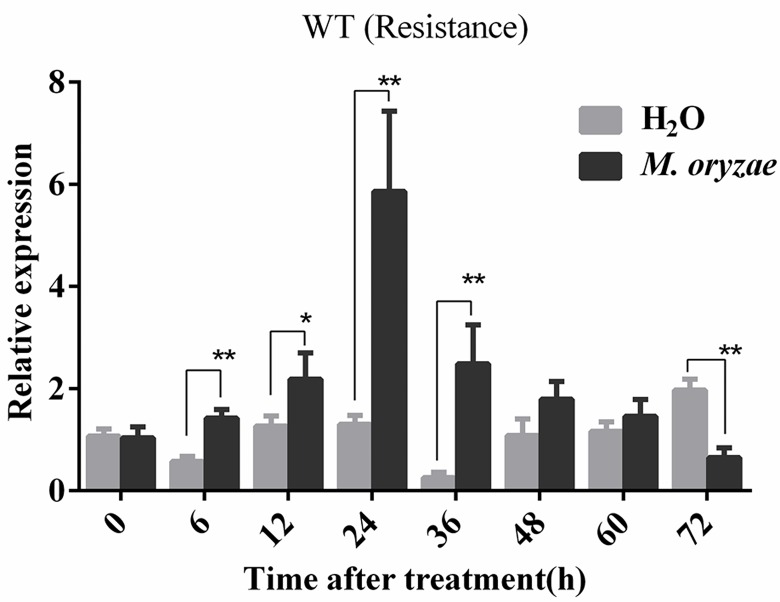
*OsCOL9* expression is up-regulated by *M*. *oryzae* infection. Relative expression of *OsCOL9* in the wild-type line at 72 h after *M*. *oryzae* inoculation. H_2_O treatment was used as control. Values shown are means±SD from three independent experiments. Error bars indicate the SD and asterisks indicate a significant difference according to the t-test (*P<0.05) compared with the control group.

We originally determined from transcriptome analysis that *OsCOL9* was a member of early response genes during *M*. *oryzae* infection (our unpublished data). Therefore, we examined the O*sCOL9* expression patterns over a time course of 72 h after *M*. *oryzae* inoculation using quantitative real-time PCR (qRT-PCR). The expression of *OsCOL9* was increased at 6 h, and it peaked at 24 h. The expression was maintained at high level to 48 h (Fig 2). These results indicated that *OsCOL9* was involved in response to *M*.*oryzae* invasion.

### Generation of *OsCOL9* transgenic plants

**Fig 3:**
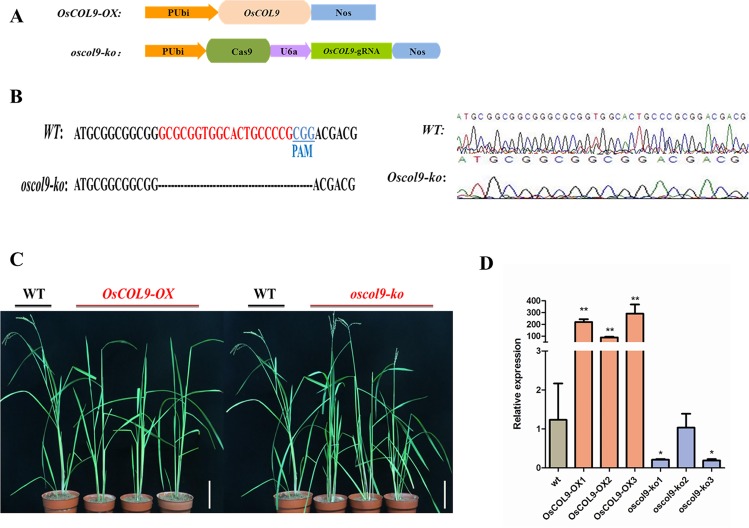
Identification of *OsCOL9* transgenic plants. (A) Schematic structure of transgenic vector construction. (B) Target sequence alignment of *OsCOL9* CRISPR/Cas9 splice sites. The blue sequence of wild-type is the PAM sequence, and the red sequence is a paired gRNA binding site compared with wild-type. Blue and red regions were spliced by the CRISPR/CAs9 endonuclease in the *OsCOL9* genome sequence. (C) Expression analysis of *OsCOL9* in over-expression and knock-out lines was measured by RT-PCR and compared with the wild-type line. Values shown are means±SD from three independent experiments (**P<0.01). (D) Morphologies of wild-type, *OsCOL9-OX* (left) and *oscol9-ko* (right). The photograph was taken 50 days after heading of transgenic plants (T1 generation) and wild-type plants. The scale bar is 8 cm.

In the present study, we attempted to obtain detailed information concerning *OsCOL9* expression during rice blast development. Therefore, we generated the *OsCOL9* over-expression and CRISPR/Cas9 knock-out plants, in which *OsCOL9* was placed under the control of the ubiquitin promoter *via Agrobacterium*-mediated transformation in wild-type plants (Fig 3A). The knock-out *OsCOL9* transgenic plants (*osclol9-ko*) were screened using target DNA sequencing (Fig 3B), and the *OsCOL9* level of transgenic rice plants *OsCOL9-OX* and *oscol9-ko* was monitored using qRT-PCR (Fig 3C and 3D). We obtained more than 10 independent *OsCOL9* over-expression transgenic plants (*OsCOL9-OX*) and one OsCOL9 CRISPR/Cas9 knock-out plant.

### *OsCOL9* knock-out plants have reduced blast resistance

**Fig 4:**
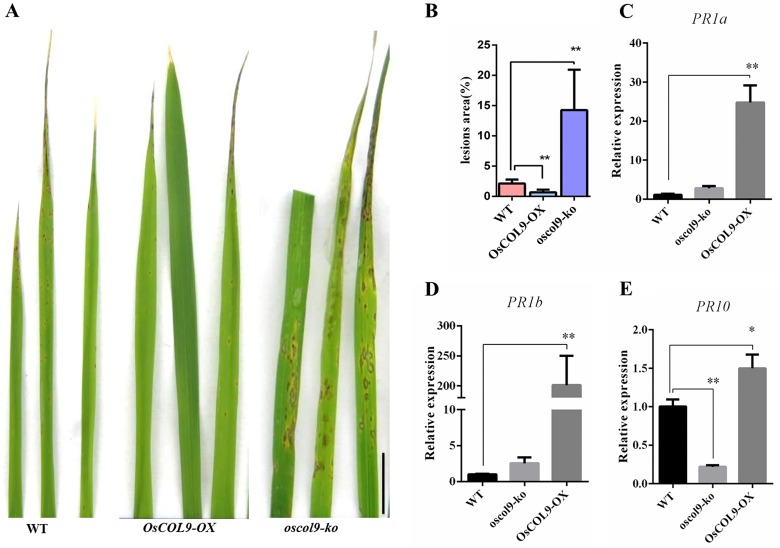
Disease resistance evaluation of *OsCOL9-OX* lines infected with the *M*. *oryzae* race GDR2. (A) A representative photograph taken at 8 days after inoculation. *OsCOL9-OX* plants showed enhanced resistance to *M*. *oryzae* race GDR2, while *oscol9-ko* became more susceptible compared with the wild-type line. (B) Lesion areas from 10 independent *OsCOL9-OX* plants. The columns shown are means±SD, and asterisks indicate a significant difference according to the t-test (*P <0.05, **P<0.01) compared with the wild-type (*Pik-H4 NILs*). (C), (D) and (E) show the expressions of *PR* genes (*PR1a*, *PR1b* and *PR10*) in wild-type, *OsCOL9-OX*, and *oscol9-ko* plants, respectively. Values shown are means±SD from three independent experiments. Error bars indicate the SD and asterisks indicate a significant difference according to the t-test (*P<0.05) compared with the wild-type plants.

To elucidate the molecular basis of *OsCOL9* in disease resistance in rice, we examined resistance of the transgenic rice lines to *M*. *oryzae* infection. Disease symptoms in plants were quantified at 8 days after inoculation. *OsCOL9-OX* lines exhibited enhanced resistance to *M*. *oryzae* as compared with the wild-type lines, while the *oscol9-ko* lines were more susceptible compared with the wild-type controls (Fig 4A and 4B). This result indicated that *OsCOL9* acted as a positive regulator of the defense response in rice.

Previous studies have shown that the appearance of disease symptoms correlates with the expressions of pathogen-related (*PR*) genes to resist *M*. *oryzae* invasion. Therefore, we determined the expressions of three *PR* genes, *PR1a* [27], *PR1b* [28] and *PR10* [29], using qRT-PCR. All three genes were up-regulated in the *OsCOL9-OX* plants as compared with wild-type and *oscol9-ko* plants under the normal growth conditions (Fig 4C, 4D and 4E). Therefore, over-expression of *OsCOL9* induced the expressions of *PR* genes in the absence of a blast infection.

### *OsCOL9* expression in response to exogenous SA and ET treatments

In the present study, we found that *OsCOL9* over-expression triggered expressions of *PR* genes, which could be associated with hormone defense responses. To determine whether *OsCOL9* was a direct regulator of hormone signaling pathways, plants were treated with phytohormones ABA, SA, JA and ACC for 48 h.

**Fig 5:**
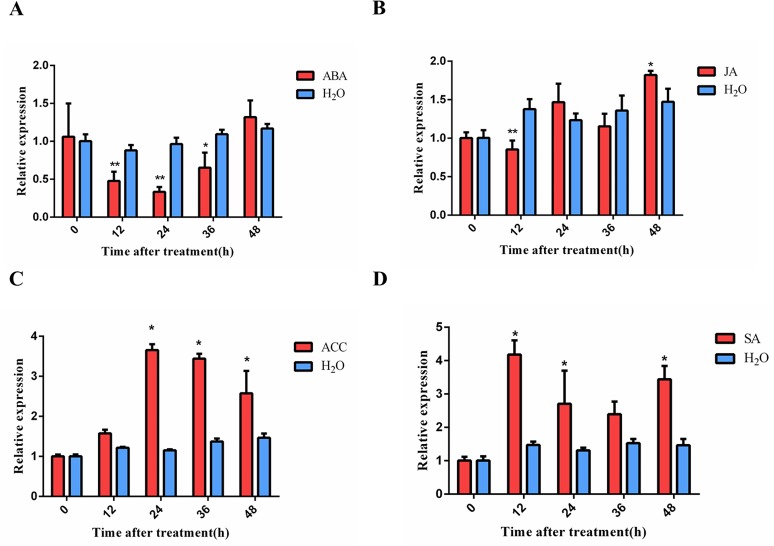
Response of *OsCOL9* to exogenous hormone treatments. (A), (B), (C) and (D) represents the expression of *OsCOL9* in fourth-leaf-stage H4 seedlings over a time course of 48 h after treated with 100 μM ABA, 100 μM JA, 1 mM ACC and 100 μM SA, respectively. H_2_O was used as control. Values shown are means±SD from three independent experiments. Error bars indicate the SD, and asterisks indicate a significant difference according to the t-test (*P<0.05) compared with the wild-type line.

The expression of *OsCOL9* at the mRNA level was increased and peaked at 12 h and 24 h after ACC and SA treatments, respectively (Fig 5). There were no significant increases after ABA or JA treatment (Fig 5). These results revealed that *OsCOL9* induced the resistance of rice against *M*. *oryzae* infection through the SA and ET signaling pathways.

### *OsCOL9* modulates the expression of phytohormone-related genes

Previous results implicated *OsCOL9* in SA- and ET-mediated defense. Because *NPR1* [30] and *WRKY45* are important modulators of *PR1a* and *PR1b* involved in the SA pathway, we determined their expression levels in *OsCOL9-OX* and *oscol9-ko* lines.

**Fig 6:**
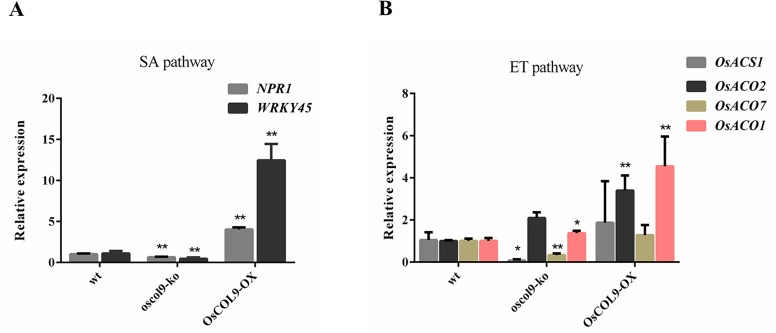
The expression of SA and ET pathway-related genes in *OsCOL9-OX* and *oscol9-ko* lines. (A) The relative expressions of *NPR1* and *WRKY45* in *OsCOL9-OX*, *oscol9-ko* and wild-type plants. (B) Gene expressions of enzymes that restrict ACC synthesis: *OsACS1*, *OsACO2*, *OsACO7* and *OsACO1*. Values shown are means±SD from three independent experiments. Error bars indicate the SD and asterisks indicate a significant difference according to the t-test (*P<0.05) compared with the wild-type plants.

*OsCOL9* over-expression modulated the expressions of *NPR1* and WRKY45 compared with the controls (Fig 6A). However, the expressions of *OsACO1* [31] and *OsACO2* [32] involved in restricting ACC synthesis significantly accumulated in *OsCOL9-OX* lines. *OsACS1* and *OsACO7* [33] did not change significantly in *OsCOL9-OX* lines, but their expressions were decreased in an *oscol9-ko* background (Fig 6B). These results demonstrated that *OsCOL9* over-expression could increase plant defense by regulating the expression of phytohormone-related genes, indicating that *OsCOL9* regulated disease resistance through the SA and ET pathways.

### OsCOL9 interacts with OsRACK1 (rice receptor for activated C kinase 1)

To illustrate the molecular mechanism of OsCOL9 in blast resistance, we performed a yeast two-hybrid assay using OsCOL9 as bait to identify the interacting proteins of OsCOL9 from a rice AD-cDNA library. Because OsCOL9 modulated transcription through its MR domain (151-300aa), we used the BB-box_1-150aa_ and CCT_301-422aa_ domains of OsCOL9 that lacked transcription activity to screen interacting proteins.

**Fig 7:**
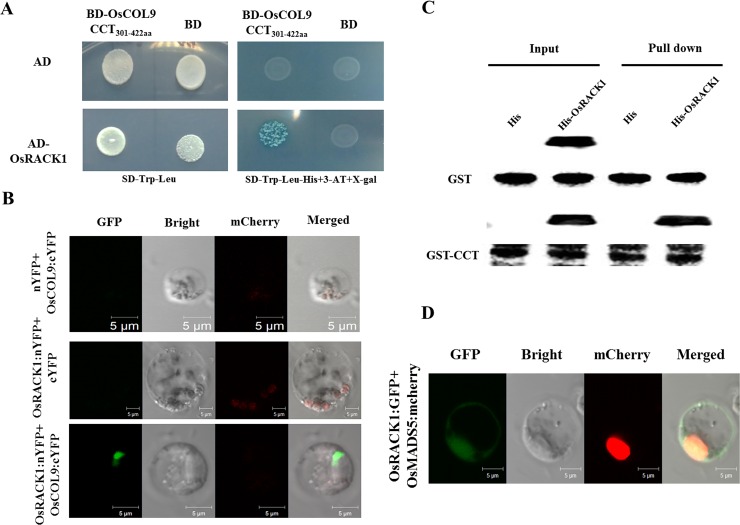
Identification of OsCOL9 and OsRACK1 interactions. (A). Yeast two-hybrid assay showed interaction between OsCOL9 CCT domain and OsRACK1. (B). Bimolecular fluorescence complementation (BiFC) assays showed the interaction between OsCOL9 and OsRACK1 in rice protoplasts. Scale bars are 5 μm. C. *In vitro* pull-down assay suggested the direct interaction between the OsCOL9 CCT domain and OsRACK1.

Yeast two-hybrid results demonstrated that CCT_301-422aa_ physically interacted with the OsRACK1 that encodes the WD40 repeat domain containing protein (Fig 7A) [34,35]. To further confirm this interaction between OsCOL9 CCT301-422aa and OsRACK1, we performed a bimolecular fluorescence complementation (BiFC) assay. The BiFC assay indicated that rice protoplast cells co-transformed with the OsRACK1:nYFP and CCT_301-422aa_:cYFP constructs displayed green fluorescence, indicating that they interacted in *vivo*, which was consistent with the Y2H assay (Fig 7B). This interaction was also observed between OsCOL9 and OsRACK1 in a GST-pull down assay in *vitro* (Fig 7C).

Furthermore, we investigated the subcellular localization of OsRACK1. Compared with the empty GFP vector, the rice protoplast cells transformed with the OsRACK1-GFP+OsMADS3-mCherry constructs displayed green fluorescence in the cytoplasm and nucleus (Fig 7D). These results suggested that OsCOL9 and OsRACK1 interacted in the nucleus.

### *OsRACK1* expression is induced by *M*. *oryzae*, SA and ACC

**Fig 8:**
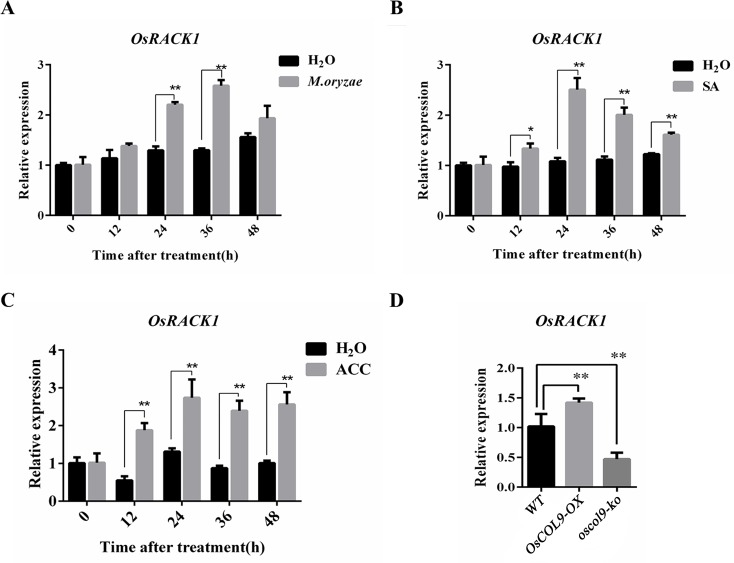
Expression of *OsRACK1* when induced by *M*. *oryzae* infection as well as SA and ACC treatments. (A), (B) and (C) represents the relative expression of *OsRACK1* treated by *M*. *oryzae*, SA and ACC after 48 h, respectively. H_2_O was used as control. Values shown are means±SD from three independent experiments. (D), Quantitative real-time PCR analyses of *OsRACK1* expression in WT and *OsCOL9* transgenic plants. Error bars indicate the SD and asterisks indicate a significant difference according to the t-test (*P<0.05) compared with the corresponding controls.

The expression of *OsRACK1* at the mRNA level can be induced by multiple plant hormones, including JA, IAA and ABA [35]. Therefore, we examined the *OsRACK1* expression in wild-type rice plants infected with *M*. *oryzae* race GDR2 or treated with the exogenous addition of SA and ACC. The expression of *OsRACK1* was increased after inoculation with *M*. *oryzae* (Fig 8A) as well as SA and ACC treatments (Fig 8B and 8C). The expression of *OsRACK1* was up-regulated in *OsCOL9* over-expression plants (Fig 8D). These data indicated that *OsCOL9* positively regulated the expression of *OsRACK1* at the mRNA level.

## Discussion

In this study, we demonstrated that OsCOL9, a COL protein, could be a pathogen-responsive transcription factor in rice. The transgenic rice plants over-expressing *OsCOL9* showed markedly enhanced resistance to blast, while *oscol9* knock-out rice plants became more susceptible (Fig 4A), indicating the importance of *OsCOL9* in rice blast resistance. Many transcription factors, such as WRKY, NAC and bZIP, play important roles in the host-pathogen interactions [36–38]. However, no COL proteins have been previously found to function in this defense pathway in rice. COL genes have major roles in photoperiodic control of flowering time [39], whereas their other roles, such as disease defense, remain unexplored.

In the present study, we described that the rice COL9 protein, OsCOL9, functioned as a pathogen-inducible transcription factor during biotic stress. Sequence analysis revealed that OsCOL9 possessed a B-box zinc finger type domain at the N-terminal region and a common C-terminal CCT domain (S1 Fig). The B-box ZF domain was assumed to be the protein–protein interaction domain, because the CCT domain is important for the nuclear localization and mediation of DNA interactions [40]. Griffiths *et al*. [41] identified OsCOL9 as a member of group II of the COL protein family. In the rice genome, there are 16 *COL* genes, suggesting that they may have other roles besides photoperiod flowering.

According to the results of the X-alpha galactosidase assay, OsCOL9 protein had transcriptional activation activity, which was mediated through its MR domain (Fig 1B). Like other transcription factors when transiently-expressed, OsCOL9-GFP fusion protein was localized to the nucleus of rice protoplast cells (Fig 1A). Furthermore, *OsCOL9* expression was significantly induced by *M*. *oryzae* in an incompatible wild-type strain, but not in a susceptible strain. This suggested that *OsCOL9* played an essential role in *R* gene-mediated resistance.

Previous studies have shown that blast resistance mediated through *Pb1* and the transcription factor *WRKY45* occurs through protein-protein interactions. Therefore, we tested whether the interaction between Pik-H4 and OsCOL9 existed, and we found that only amino acids 1–266 of the Pik_1_-H4 protein interacted with OsCOL9 (S2 Fig). Previous studies have also indicated that Pik_1_-H4 can be localized in the nucleus [42]. Therefore, the defense signals derived from the recognition between Pik_1_-H4 and Avr-Pik may transfer the signals downstream depending on OsCOL9. In addition, over-expression of *OsCOL9* could delay the flowering time, suggesting that *OsCOL9* mediated cross-talk between flowering time and blast resistance in rice.

Fig 5C and 5D illustrate that *OsCOL9* was sensitive to exogenous SA or ET treatment in the incompatible rice *Pik-H4 NILs* (WT), suggesting a role of *OsCOL9* in the SA- and ET-related signaling pathways. Most transcription factors play crucial roles in the regulation of hormone signaling pathways. For example, *AtWRKY53* and *AtWRKY70* play a positive role in SA-mediated resistance to *P*. *syringae* infection [43,44], and *OsWRKY45* acts in the SA signaling pathway independently of *NPR1*. Our results showed that the expression levels of *NPR1* and *WRKY45* were reduced in *oscol9-ko* plants, indicating that *OsCOL9* possibly acted upstream of *NPR1* (Fig 6A). The expressions of the *PR* genes *PR1a* and *PR1b* were high in *OsCOL9-OX* lines, implying that the expressions of these two genes were dependent on *OsCOL9*. These data indicated that *OsCOL9* acted in SA-mediated resistance to *M*.*oryzae* upstream of *NH1*, *PR1*a and *PR1b*.

ET has been also implicated as a vital player in regulation of defense networks by activating or suppressing the expressions of downstream defense genes [45]. Over-expression of transcription factors of the ERF subfamily, such as *AtERF2*, *AtERF14* and *OsBIERF3*, enhances resistance to several pathogens by regulating transcription of defense genes [46–48]. Our results showed that the *OsCOL9* expression could be significantly induced by exogenous addition of the ET precursor ACC. ACC synthase (ACS) and ACC oxidase (ACO) genes contribute to ET biosynthesis in blast fungus-infected rice plants [49], and the expression levels of these genes are increased. Compared with the wild-type plants, the expressions of *OsACO1* and *OsACO2* were significantly increased in *OsCOL9-OX* lines, while the expressions of *OsACO7* and *OsACS1* were decreased in *oscol9-ko* plants. Such different expression patterns in these two different transgenic rice plants suggested a complicated role of *OsCOL9* in the regulation of ET biosynthesis. These results indicated that the *OsCOL9* most likely acted in defense pathways through indirect regulation of ET synthesis.

OsRACK1 has been previously reported as an interacting partner of Rac1 that modulates blast resistance and is involved in ABA and H_2_O_2_ signaling pathways to regulate seed germination. The OsRACK1 protein contains a seven WD40-repeat domain that is highly conserved in eukaryotic organisms, and it contains several tandemly repeated subunits that function in multiple pathways [50]. Our results suggested that OsCOL9 was associated with OsRACK1 to enhance rice blast resistance, which was most likely involved in SA and ET signaling pathways. Previous studies have determined that the WD40-containing protein DACF acts as a substrate-recognition receptor for CULLIN4-based E3 ubiquitin ligases, and it interacts with DDB1 to mediate the COP9 signalosome degradation in *Arabidopsis thaliana* [51]. Therefore, we thought that OsRACK1 interacts with OsCOL9 and probably recruits E3 ligase complex to mediate degradation *via* the ubiquitin proteasome system [52]. There is clear evidence that RACK1 induces the RANKL-dependent activation of p38 MAPK ubiquitin pathway, which is in agreement with our hypothesis [53]. However, the mechanism of *OsRACK1* up-regulation in *OsCOL9* transgenic plants remains still unclear.

In conclusion, our results suggested that *OsCOL9* could control the blast resistance through the regulation of SA- and ET-mediated signaling pathways in rice. Previous studies have indicated that most *COL* genes exhibit diverse roles, including flowering, circadian rhythm and root growth, in plant development [54,55]. Therefore, it is necessary to determine the precise function of *OsCOL9* during plant development in future studies. Furthermore, the analysis of *OsCOL9*-induced crosstalk between the disease resistance and plant development will offer a novel insight into COL-protein-mediated physiological and biochemical response in rice.

## Supporting Information

S1 Fig Exon/intron structure of *OsCOL9*, alignment and phylogenetic analysis of *OsCOL9* with other COL amino acid sequences. (PDF)

S2 Fig Physical interactions between the Pik-H4 and OsCOL9 detected in yeast two-hybrid assay. (PDF)

S3 Fig Expression patterns of *OsCOL9* in different plant tissues. (PDF)

S4 Fig ABA and JA synthetic l gene expression in *OsCOL9* transgenic and wildtype plants. (PDF)

S5 Fig Relative expression of *OsCOL9* in the susceptible cultivar LTH at 72 h after *M*. *oryzae* inoculation. (PDF)

S1 Table Primers used in vector construction. (PDF)

S2 Table Primers used in qRT-PCR. (PDF)
