# Supplementary material for: CONSTANS-Like 9 (OsCOL9) Interacts with Receptor for Activated C-Kinase 1(OsRACK1) to Regulate Blast Resistance through Salicylic Acid and Ethylene Signaling Pathways
Source: PLoS One. 2016 Nov 9;11(11):e0166249. doi: 10.1371/journal.pone.0166249 (PMC5102437; doi:10.1371/journal.pone.0166249)
Supplement: S1 Fig — (PDF) [file pone.0166249.s001.pdf]

A

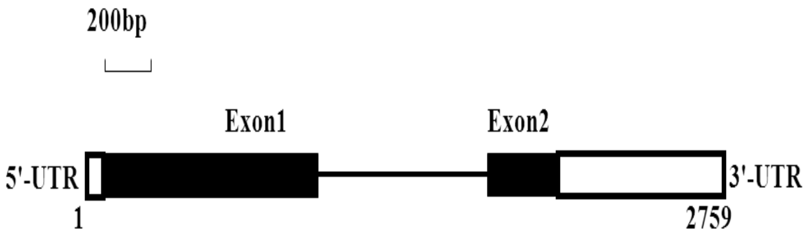
200bp
Exon1
Exon2
5'-UTR
3'-UTR
1
2759

B

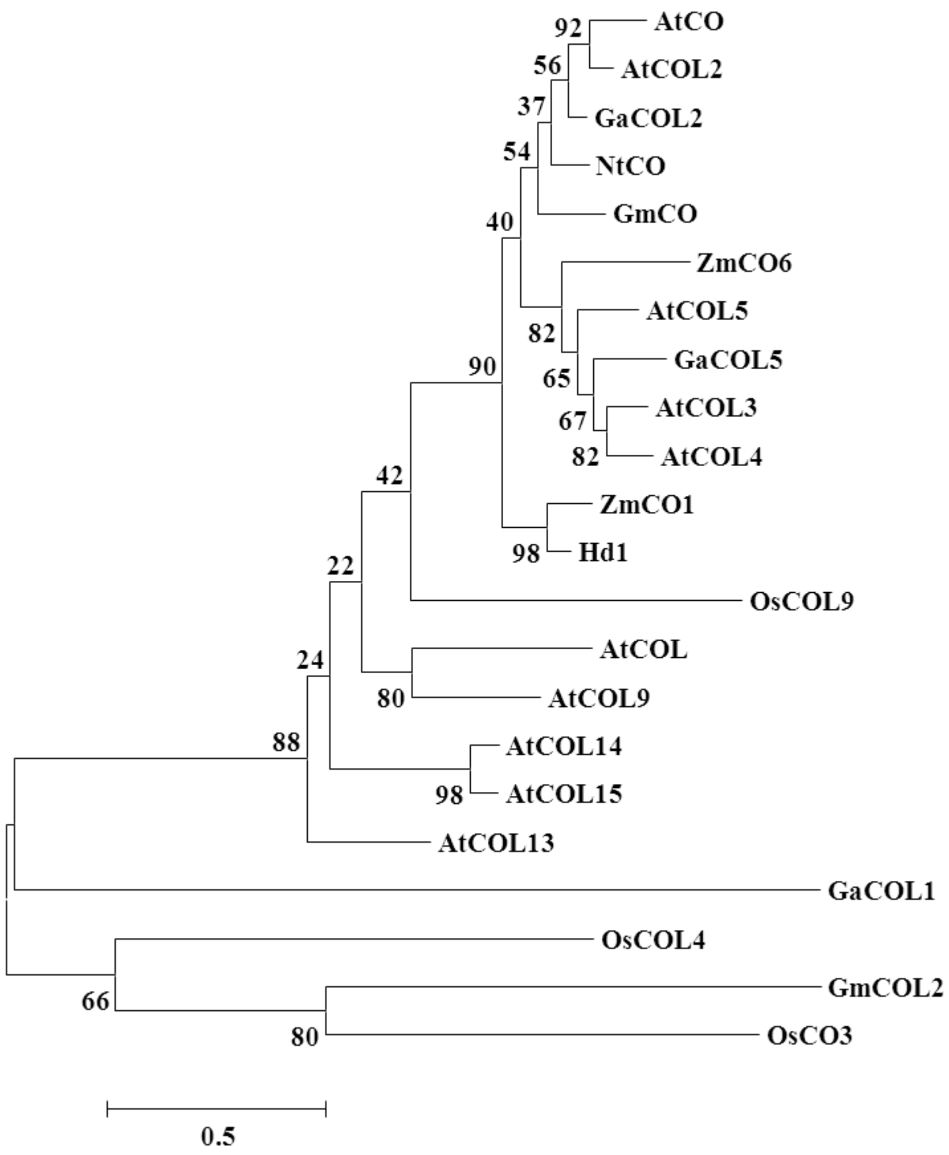
AtCO
AtCOL2
GaCOL2
NtCO
GmCO
ZmCO6
AtCOL5
GaCOL5
AtCOL3
AtCOL4
ZmCO1
Hd1
OsCOL9
AtCOL
AtCOL9
AtCOL14
AtCOL15
AtCOL13
GaCOL1
OsCOL4
GmCOL2
OsCO3
92
56
37
54
40
82
65
67
82
90
42
98
22
24
80
88
98
66
80
0.5

C

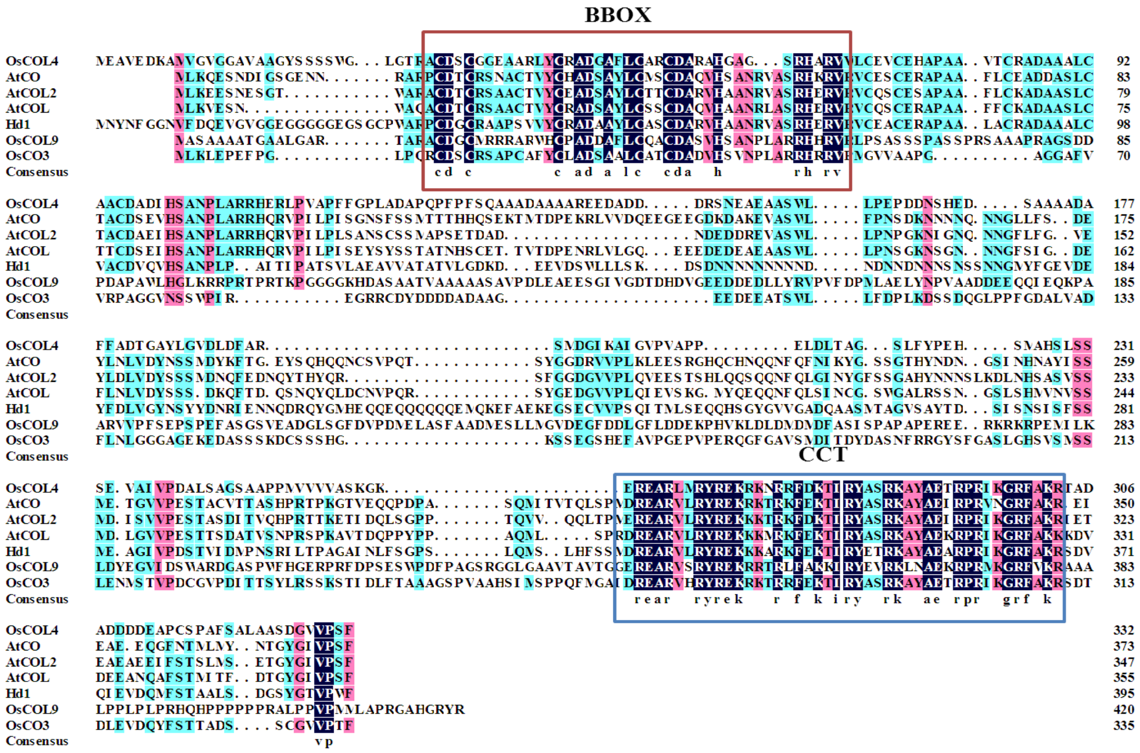

S1 Fig. Exon/intron structure of OsCOL9 and alignment and phylogenetic analysis of OsCOL9 with other COL amino acid sequences. (A) OsCOL9 has two exons (boxes) and one intron (line between the two boxes). Closed boxes indicate coding region, and open boxes show the 5’ and 3’ untranslated regions (UTR). (B) Phylogenetic relationships of OsCOL9 proteins and selected species with the MEGA6 program using the Neighbor-Joining method. The genes and accession numbers were AtCO (CAA64407), AtCOL2 (AAB67880), GaCOL2 (KHG24977), NtCO (AEJ84000), GmCO (ACJ65311), ZmCO6 (NP001148229), AtCOL5 (Q9FHH8), GaCOL5 (KHG29079), AtCOL3 (Q9SK53), AtCOL4 (Q940T9), ZmCO1 (NP001120722), Hd1(AB041837), OsCOL9 (LOC_Os03g50310), AtCOL (CAB78567), AtCOL9 (AEE74583), AtCOL14 (O22800), AtCOL15 (Q9C78E), AtCOL3 (O822256), GaCOL1 (KHG24445), OsCOL4 (AK100097), GmCOL2 (NP001278944), OsCO3 (AB00187). (C) Amino acid sequence alignment of OsCOL9 and other COLs from rice and Arabidopsis. Their gene accession numbers have been showed in (B). The N-terminal conserved domain B-box was highlight marked by red rectangle. The C-terminal CCT domain was highlight marked by blue rectangle.
