# Supplementary material for: CONSTANS-Like 9 (OsCOL9) Interacts with Receptor for Activated C-Kinase 1(OsRACK1) to Regulate Blast Resistance through Salicylic Acid and Ethylene Signaling Pathways
Source: PLoS One. 2016 Nov 9;11(11):e0166249. doi: 10.1371/journal.pone.0166249 (PMC5102437; doi:10.1371/journal.pone.0166249)
Supplement: S2 Fig — (PDF) [file pone.0166249.s002.pdf]

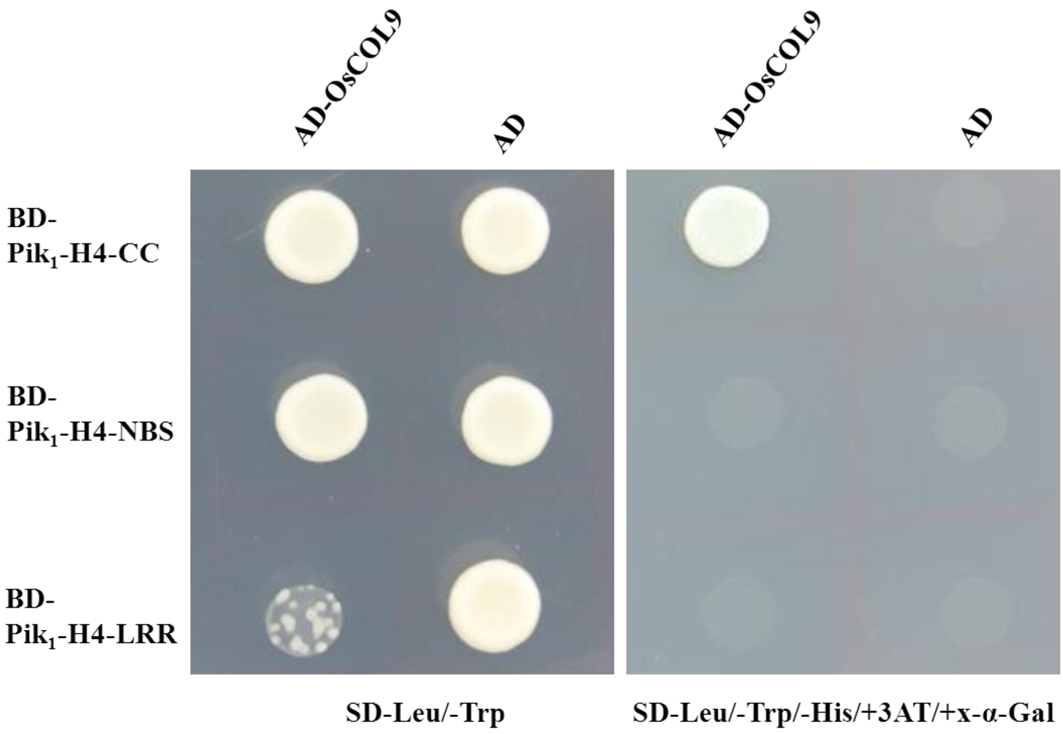

S2 Fig. The physical interaction between the Pik-H4 and OsCOL9 detected in yeast two-hybrid assay. The yeast strain AH109 gold cells were transformed with plasmid: BD-Pik1-H4+AD-OsCOL9, which grow well on SD/-Trp/-Leu and SD/-Trp/-Leu /-His/+3AT/+x-α-Gal agar plate and this result can be considered positive for interaction.
