# Supplementary material for: CONSTANS-Like 9 (OsCOL9) Interacts with Receptor for Activated C-Kinase 1(OsRACK1) to Regulate Blast Resistance through Salicylic Acid and Ethylene Signaling Pathways
Source: PLoS One. 2016 Nov 9;11(11):e0166249. doi: 10.1371/journal.pone.0166249 (PMC5102437; doi:10.1371/journal.pone.0166249)
Supplement: S3 Fig — (PDF) [file pone.0166249.s003.pdf]

A

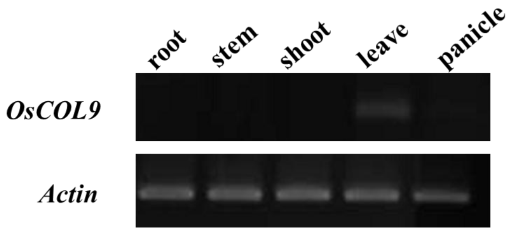

B

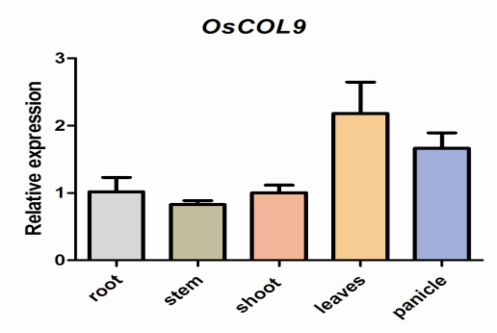

S3 Fig. The expression patterns of OsCOL9 in different tissues. (A) Semi-quantitative suggested that OsCOL9 significantly expressed in leaves and weakly expressed in panicle. (B)The results of quantitative PCR analysis is consistent with semi-quantitative.
