# Supplementary material for: CONSTANS-Like 9 (OsCOL9) Interacts with Receptor for Activated C-Kinase 1(OsRACK1) to Regulate Blast Resistance through Salicylic Acid and Ethylene Signaling Pathways
Source: PLoS One. 2016 Nov 9;11(11):e0166249. doi: 10.1371/journal.pone.0166249 (PMC5102437; doi:10.1371/journal.pone.0166249)
Supplement: S4 Fig — (PDF) [file pone.0166249.s004.pdf]

A

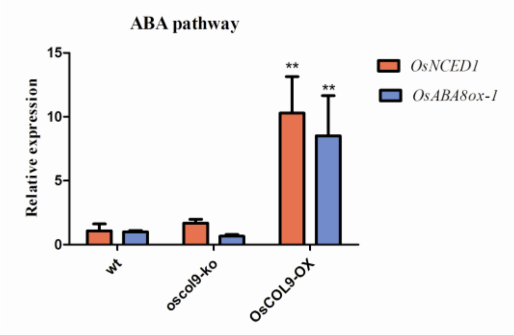

B

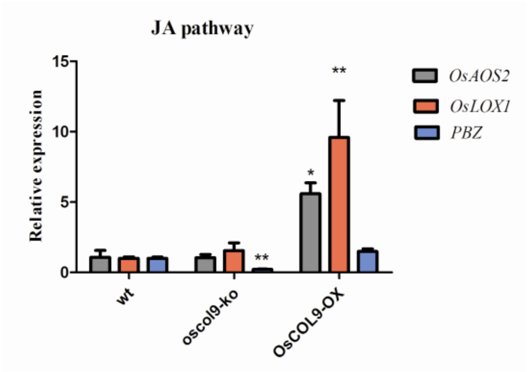

S4 Fig. ABA and JA synthesis critical genes expression in OsCOL9 transgenic plants and wild-type.(A) OsNCDE1 and OsABA8ox-1 expression significantly increased in OsCOL6ox, both of them involved in ABA synthesis pathway.(B)The expression of OsAOS2, OsLOX1, and PBZ in OsCOL9 transgenic plants and wild-type, which were reported associated with JA synthesis. Values shown are means ±SD from three independent experiments. Error bars indicate the SD and asterisks indicate a significant difference according to the t-test ($P < 0.05$) compared with the corresponding controls.
