# Supplementary material for: CONSTANS-Like 9 (OsCOL9) Interacts with Receptor for Activated C-Kinase 1(OsRACK1) to Regulate Blast Resistance through Salicylic Acid and Ethylene Signaling Pathways
Source: PLoS One. 2016 Nov 9;11(11):e0166249. doi: 10.1371/journal.pone.0166249 (PMC5102437; doi:10.1371/journal.pone.0166249)
Supplement: S5 Fig — (PDF) [file pone.0166249.s005.pdf]

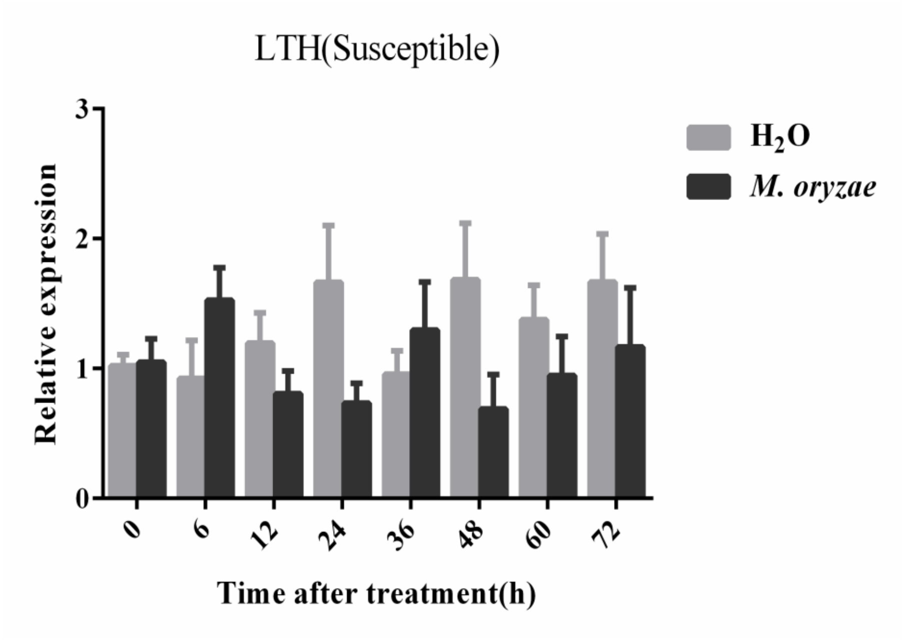

Fig. S5 Relative expression of OsCOL9 in the susceptible cultivar LTH at 72 h after *M. oryzae* inoculation.
