# Supplementary material for: CONSTANS-Like 9 (OsCOL9) Interacts with Receptor for Activated C-Kinase 1(OsRACK1) to Regulate Blast Resistance through Salicylic Acid and Ethylene Signaling Pathways
Source: PLoS One. 2016 Nov 9;11(11):e0166249. doi: 10.1371/journal.pone.0166249 (PMC5102437; doi:10.1371/journal.pone.0166249)
Supplement: S1 Table — (PDF) [file pone.0166249.s006.pdf]

S1 Table. Primers used in various vectors construction

Primer Name	Sequence
pGBKT7	
OsCOL9 Y2H-F	GTTGTTCATATGATGGCGAGCGCCGCCGCGGCGACG
OsCOL9 Y2H-R	GTTGTTGGATCCTCAAAAACGGTAGCGCCCGTGCGC
Subcellular localizatior	
OsCOL9 GFP-F	GTTGTTCATATGATGGCGAGCGCCGCCGCGGCGACG
OsCOL9 GFP-R	GTTGTTGGATCCAAAACGGTAGCGCCCGTGCGC
Transcription activity	
BBOX Nde-F	GTTGTTCATATGATGGCGAGCGCCGCCGCGGCG
BBOX Bamh-R	GTTGTTGGATCCGTCGTCCTCTTCCCCGACGTC
MR Nde-F	GTTGTTCATATGGAGGACCTGCTGTACCGCGTC
MR Bamh-R	GTTGTTGGATCCCGGCGAGGCTCCGTCGCGGGC
CCT Nde-F	GTTGTTCATATGTGGTTCCACGGCGAGCGCCCT
CCT Bamh-R	GTTGTTGGATCCTCAAAAACGGTAGCGCCCGTG
Transgenic plants	
OsCOL9 ox-F	GTTGTTGGATCCATGGCGAGCGCCGCCGCGGCGACG
OsCOL9 ox-R	GTTGTTGGATCCTCAAAAACGGTAGCGCCCGTGCGC
OsCOL9 U6a-F	GCCGCGCGGTGGCACTGCCCCG
OsCOL9 U6a-R	AAACCGGGGCAGTGCCACCGCGC
OsCOL9 cas-F	ACACTACTGTTCCCATTTTTT
OsCOL9 cas-R	TTCTGCTCGATCTGCTGCT
Hpt-F	TCCGGAGCCTCCGCTCGAAGTAG
Hpt-R	CTGAACTCACCGCGACGTCTGTC
For interaction assay	
ADOsCOL9Nde-F	GTTGTTCATATGGCGGGAAGCCGGGGAGGGCTCGG
ADOsCOL9BamH-R	GTTGTTGGATCCCCGCTTCTCGGCGTTGAGCTTGC
Pik1-H4CC Y2H-F	GAATTCATGGAGGCGCTGGCCATGGCCGTA
Pik1-H4CC Y2H-R	GGATCCCTAAAATTCACATATGGATTTCACCGGC
Pik1-H4NBS-F	GAATTCCACAAGGTCAAAACAGTTTGCA
Pik1-H4 NBS-R	GGATCCCTAGGAGGATGCACTAGTACTAG
Pik1-H4 LRR-F	GAATTCCCAAGGTTGATTCGCCGGCT
Pik1-H4 LRR-R	GAATTCCTAGGCGCTGATGCCAGGGGCGTCGCTCTT
RACK1 Nde-F	GTTGTTCATATGATGGCCGGCGCGCAGGAGTC
RACK1 BamH-R	GTTGTTGGATCCCTAGCCGGCGTAGCTGAAACC
RACK Nhe-F	GCTAGCATGGCCGGCGCGCAGGAGTC
RACK1 Age-R	ACCGGTCTAGCCGGCGTAGCTGAAACC
RACK1 BamH-F	GGATCCATGGCCGGCGCGCAGGAGTC
RACK1 EcoR-R	GAATTCCTAGCCGGCGTAGCTGAAACC
OsCOL9 Nhe-F	GCTAGCATGGCGAGCGCCGCCGCGGCG
OsCOL9 Age-R	ACCGGTTCAAAAACGGTAGCGCCCGTG
CCT BamH-F	GGATCCTGGTTCCACGGCGAGCGCCCT
CCT EcoR-R	GAATTCTCAAAAACGGTAGCGCCCGTG
