# Supplementary material for: CONSTANS-Like 9 (OsCOL9) Interacts with Receptor for Activated C-Kinase 1(OsRACK1) to Regulate Blast Resistance through Salicylic Acid and Ethylene Signaling Pathways
Source: PLoS One. 2016 Nov 9;11(11):e0166249. doi: 10.1371/journal.pone.0166249 (PMC5102437; doi:10.1371/journal.pone.0166249)
Supplement: S2 Table — (PDF) [file pone.0166249.s007.pdf]

S2 Table. Primers used in RT-PCR

Primer Name	Sequence
RT-PCR	
OsCOL9-F	CGATCCCAGCGAGTCATG
OsCOL9-R	GATCTTCTTGGCGAACAG
PBZ-F	AAGTCGGATGTGCTCGAGG
PBZ-R	GATGTCCTTCTCCTTCTCC
PR1a-F	AAGCTGGAGCACTCGGACT
PR1a-R	ACACCACCTGCGTGTAGTG
PR1b-F	ACTGGACGGCGGCGAGCGCG
PR1b-R	CTTATAGTTGCATGTGATG
NPR1-F	AGCTCGGATGACGGCACTC
NPR1-R	CTTCTGAAGAACATCCTGCA
OsLOX1-F	AAGAACTGGAAGTTCACCGAG
OsLOX1-R	GATGGCCAGGTACTCGCCCAC
OsAOS2-F	AGCTAGCTAGAAGAGAGTTAGC
OsAOS2-R	ACTCGAAGTACTTGTCCTGC
OsACO1-F	CTGCGGCGATGGAGCAGCTGGA
OsACO1-R	CACGAACTTGGGGTACGCCACGA
OsACS1-F	TCGGCCAAGACCCTCGACG
OsACS1-R	CGAAAGGAATCTGCTACTGCTGC
OsACO7-F	GTGATCGCGCCGGCGACGGC
OsACO7-R	GGGGAACCCTGCGTACTAC
OsACO2-F	AGCAACCCCGGCCTCGCTC
OsACO2-R	AGGGACTTGCTATGACACGG
OsNCED1-F	CTCACCATGAAGTCCATGAGGCTT
OsNCED1-R	GTTCTCGTAGTCTTGGTCTTGGCT
OsABA8ox1-F	CCAAGAACCCCAACGTGTTC
OsABA8ox1-R	CGGGCTGGACACCATCA
Ubq-F	ACCCTGGCTGACTACAACATC
Ubq-R	AGTTGACAGCCCTAGGGTG
RACK1-F	TCAAGGTGTGGAACCTCACGAA
RACK1-R	CAGAGCGAGTGAATGATGGAA
